# Letter from Williamsburg

**Published:** 1861-10

**Authors:** J. G. Westmoreland


Vol. VIII.] OCTOBER, 1861. [No. 2.

### ORIGINAL COMMUNICATIONS.

### ARTICLE II.

College Hospital, Williamsburg, Va., Sept. 21, 1861.

*Dear Dr. Brown:*

The long promised communication is slow to come, nor do I know whether the old adage, "better once than never," would properly apply to this article.

Since arriving in Virginia, I have seen and heard many interesting things in Medicine and Surgery. My first week was spent as most strangers in Richmond spend theirs before going into camp—in seeing what was going on. Being then only two or three weeks after the great battle of Manassas, many important surgical cases were to be seen, of which to some extent you were informed in my first letter. Of my second week in and around the temporary capital of the Confederate States, nothing can be said that would interest a medical man. The stern realities of army life, without much sickness in our camp, but with the systematical thumping of drums, blast of bugles hour after hour, till they became as monotonous as the screeching of the July-fly of an evening in Georgia. Once in a while, but rarely, the roar of cannon by artillery companies in our neighborhood, drilling, roused us to the reflection that we were there preparing to destroy the cowardly invaders of our soil.

I was transferred to Hospital service three weeks since, and from that time to the present, have constantly, in my waking hours, witnessed the devastation resulting from the collection of armies of men, in moist, marshy districts, during the warm season.

In order the more readily to trace the cause leading to the various diseases with which the division of the army in this neighborhood is affected, something of its topography becomes necessary. Williamsburg, the point from which I write, and the location of the General Hospital for (and a most extensive one) Gen. Magruder's army, is situated midway between the James and York rivers. The point of land lying between these rivers is, at this point, six miles across, and on it, scattered for thirty miles between these streams, is Gen. Magruder's command, or, as it is here called, the Army of the Peninsula. The face of the country is level, low, damp, and in many places contiguous to the rivers, interspersed with ponds and marshes. For the most part, low-country soldiers are found in the army here. Several Louisiana regiments, and the Zouave battalion from New Orleans, with several companies from lower Georgia, are among them. This would seem to have been a judicious and satisfactory arrangement; but it is a fact I have had sufficient evidences to sustain, that the Louisiana troops, particularly the Zouaves, from the city of New Orleans, suffer more from malaria than any other portion of the army. It would seem that those accustomed to low, marshy country, where malaria abounds, would be less likely to suffer here, and why it is not so, is explained only in the fact that these who were suppo sed to be more inured to malaria were purposely, or accidentally, placed at the most sickly locations. While few of them have escaped an attack of fever, there has been decidedly less mortality among them than others owing to the fact that remittent fever rarely proves fatal, and typhoid fever rarely attacks those exposed to malaria constantly. It is true, we have both forms of fever in the same regiment frequently, and more intimately connected with each other than I have before witnessed, yet some companies of a regiment on the Peninsula are frequently exposed to influences which some others of the same regiment escape altogether. The summit of a hill, for instance, overlooking a marsh, would be more likely to expose troops to malarial influence than a less elevated situation though even nearer the point where the miasm is generated.

Hence it is that those living on the low lands of streams, in what is sometimes supposed to be very sickly localities, are less liable to intermittent and remittent fevers than their neighbors who seek elevated localities where pure water and cool breezes are enjoyed. The history of malarious districts proves conclusively that miasm ascends from the place of its generation, to a certain height, and then passes horizontally in whatever direction the current of air may chance to carry it, infecting the elevated positions lying in its course. While the marshes and ponds on this peninsula are prolific of the intermittent and remittent agent, unfortunately for the soldier, those points not thus exposed are infected with the cause of a more serious malady, typhoid fever. This, though in the main, not of a malignant character, runs its usual tedious and prostrating course, and to the soldier, deprived, while in camp, of the most suitable diet and attention, often proves fatal, when under other and more favorable circumstances it might not.

Very embarrassing circumstances surround all cases of serious disease in this region of country, during the first month or two. I have never seen a warmer September, even in Georgia, and this, with the constant dampness from the frequent rains and flat situation of the surface of the country, leads to the two great prevailing complications, diarrhœa and bronchitis. It is rarely that a case goes through with either of the forms of fever without one of these difficulties, and in many instances, in the very onset of the disease, both of these troublesome complications are developed. Aperients are rarely indeed called for in any form of fever. It is an exception to the general state of things when a case with constipation of the bowels is found. And in passing, by way of parenthesis, as one of my best friends is wont to speak, I would say to those in the habit of using aperients and purgatives in fever preceding the abortive means, that this part of their usual treatment is entirely superceded by the natural influences here exerted. Bronchitis, sometimes running rapidly into irritation and engorgement of the perynchema of the lungs, is very common, not only in continued or typhoid fever, but often is very annoying in the cases affected with remittent and intermittent fever. A blister to the upper front of the chest I have found, for several years, the best means of arresting this sometimes very serious difficulty in typhoid fever, and have had every reason to be pleased with that treatment here. No other means that I have seen used seems to act so well; sedatives, expectorants, &c., particularly the former, seem inappropriate in a disease evidently odynamic in its character and tendencies, and experience in the use of those means most useful in pulmonary disease with a sthenic state of the system, proves that they are at best ineffectual, while counter irritation has no depressing influence (always to be avoided in typhoid fever) and is known to afford relief. The same difficulty is not met with in remittent fever. No danger need be apprehended usually from bronchial irritation in this disease, and may be allayed usually with opium and other soothing remedies.

Diarrhœa, so very common in fevers of all kinds here, does not lead to such amount of prostration and consequent danger in typhoid fever as is generally observed in Georgia. This is, perhaps, explained in the fact that while it usually depends upon actual lesions in the bowels, many cases here depend, perhaps, on simply an exhalation of serum into the canal, without any serious disturbance otherwise. Astringents of the vegetable kind, opiates, &c., generally restrain the discharges, particularly when used by the rectum and stomach.

The distinguishing symptoms of typhoid and remittent or intermittent fevers are to be found here as plainly, in recent cases, as observed in the neighborhood of Atlanta. A difficulty however exists in not being able to see most of the cases until after a week or more has elapsed from the attack. The character, symptoms of uneasiness, tenderness and pain in the region of the medulla, is generally obscure, or passes entirely away within that time; and in several instances the test of full doses of quinine gives reliable evidence of the character of fever under treatment. So also, with the pain and tenderness so constant in recent cases of remittent and intermittent fever felt in the dorsal and lumbar region. These symptoms have passed away in many cases before entering the hospital. In some cases of each, however, they are very distinct at the time of admission, and prove, as usual, evidences almost amounting to pathognomonic.

In the general treatment of fevers, typhoid and remittent, or intermittent, I have met with no reason to change that which, in Atlanta, we are accustomed to—the supporting, stimulating and soothing treatment for the former, and the anti-periodic or nervous tonic treatment for the latter, succeeds very well. I am testing thoroughly the efficacy of blistering over the region of the medulla. In one ward, I have ten cases of typhoid fever. Some of them were blistered early after admission, others only when very unfavorable symptoms connected with the nervous system were exhibited, and some others have had no blister at all, or failed to have thorough vesication. There are to be found in this ward unmistakable evidences of the utility of blistering at this point in typhoid fever. The same may be said of the benefit of nourishing fluid diet and alcoholic drinks given regularly and in full quantities.

The preparatory treatment of remittent and intermittent fever, to the use of quinine, we dispense with entirely, for two reasons, either of which would be sufficient; and 1st, patients sent here have generally undergone all that the strongest advocate for "*puking and purging*" in fever, could desire; and 2dly, because no advantage whatever can accrue to the patient from delaying the administration of that which will, with a great deal of certainty, prevent the approaching paroxysm of fever and arrest its progress entirely, for the sake of imaginary good in giving greater certainty to the action of the nervous tonic. Whole wards are relieved, cured, in three days, without any "*cleansing of the stomach*" heretofore so much talked of.

Malignant intermittent fever, sometimes called congestive fever, is brought in occasionally, and requires promptly the bold administration of quinine and alcoholic drinks.

The difficulty met with, is the deceptive character of the disease. It is generally almost perfectly intermittent in form, and when seen only during the intermission, would not be looked upon as alarming, but which may terminate, without counteracting remedies, in a few hours, in perfect prostration of the vital energies, with fatal congestion.

Of dysentery, I have not seen more than a dozen cases, and perhaps none of them have exhibited a very marked degree of malignancy; yet the usual tendency to low grade of fever found in epidemic dysentery in our State. Mercurials have very little influence for good. Nourishing and supporting treatment in the main seems to answer best, with fluid diet of nourishing quality, wine, opium, &c. We meet with cases, however, which yield readily to full doses of sulph. magnesia and tinct. opii. Astringent and opiate injections and blisters to the hypogastrium are of immense value.

I have not as yet met with in the hospital anything of surgery worth mentioning; only the ordinary casualties of an army having no engagement in battle. Should the enemy attempt an invasion of the peninsula, we shall, as at Manassas, have many of his, as well as our own, to care for in surgical wards.

Of the whole number now in the wards, say 190, about one-fifth have typhoid fever, one fifth remittent fever, two fifths intermittent fever, and one fifth various other diseases, wounds, &c. The mortality in the Hospital, from its opening to the present, is less than three per cent.—perhaps but little above two per cent. The per centage is, no doubt, smaller on account of the preponderance of intermittent fever over the more violent and dangerous forms of disease. With the amount of typhoid fever, dysentery and pneumonia, admitted during the summer, it is as small proportion of deaths as is generally met with anywhere. Truly,

J. G. WESTMORELAND.

